# Predicting the acute pancreatitis severity with multi-machine learning models: constructing an online prediction platform

**DOI:** 10.3389/fcimb.2026.1760036

**Published:** 2026-02-27

**Authors:** Jie Cao, Shike Long, Huan Liu, Ribin Liao, Fu,an Chen, Xiyou Li, Lifeng Xu, Ying Liu

**Affiliations:** 1 Department of Gastroenterology, The Second Affiliated Hospital of Guilin Medical University, Guilin, Guangxi, China; 2 School of Aeronautics and Astronautics, Guilin University of Aerospace technology, Guilin, Guangxi, China; 3 Guangxi Key Laboratory of Low-Altitude Unmanned Autonomous Systems, Guilin, Guangxi, China; 4 Guangxi Health Commission Key Laboratory of Glucose and Lipid Metabolism Disorders, The Second Affiliated Hospital of Guilin Medical University, Guilin, Guangxi, China; 5 Guangxi Key Laboratory of Metabolic Reprogramming and Intelligent Medical Engineering for Chronic Diseases, The Second Affiliated Hospital of Guilin Medical University, Guilin, Guangxi, China

**Keywords:** LightGBM, machine learning, online prediction platform, predictive models, severe acute pancreatitis

## Abstract

**Background:**

Early assessment of acute pancreatitis (AP) severity is critical. We therefore built a web-based calculator that instantly estimates the probability that a patient admitted with AP will progress to the severe form.

**Methods:**

Clinical records for patients who were diagnosed as AP at the Second Affiliated Hospital of Guilin Medical University between the start of 2016 and May 2025 were retrospectively examined. The dataset was randomly divided into training set (70%) and test set (30%). For the traditional machine learning models, we employed 5-fold cross-validation combined with random search for hyperparameter optimization during training. Feature selection was performed using Random Forest (RF) and the Least Absolute Shrinkage and Selection Operator (LASSO) methods. Model construction included Logistic Regression (LR), Decision Tree (DT), Naive Bayes (NB), Support Vector Machine (SVM), Multi-Layer Perceptron (MLP), Light Gradient Boosting Machine (LightGBM), Extreme Gradient Boosting (XGBoost), Artificial Neural Network (ANN), Convolutional Neural Network (CNN), and Long Short-Term Memory Network (LSTM). The area under the receiver operating characteristic curve (AUC), among other metrics, served to evaluate model efficacy. SHapley Additive exPlanations (SHAP) and Partial Dependency Plots (PDP) were employed to explain model predictions, and a clinical application risk prediction platform was further developed.

**Results:**

1289 patients with AP were included, with 11 variables screened to develop 10 models. Among these, the LightGBM demonstrated the highest predictive accuracy on training and test sets, with AUC (95% CI) values of 0.9726 (0.9626-0.9818) and 0.9301 (0.9113-0.9481), respectively. SHAP and PDP analyses identified Ca, WBC, α-HBDH, and Glu as key predictive features for severe acute pancreatitis (SAP). Calcium levels exerted a negative influence on SAP prediction, whereas WBC, α-HBDH, and Glu exerted positive influences, exhibiting positive synergistic effects among these three variables.

**Conclusion:**

Our study highlights the substantial predictive potential of Ca, WBC, α-HBDH, and Glu for SAP. We have built a predictive online platform for clinical use, enabling healthcare professionals to rapidly and effectively assess SAP risk, thereby facilitating timely intervention and treatment.

## 1 Introduction

Acute pancreatitis (AP) is a common gastrointestinal disorder with a global incidence rate of approximately 30–40 per 100,000 individuals (Szatmary et al., 2022). Approximately 20-40% of AP cases progress to severe acute pancreatitis (SAP). Once SAP develops, it is associated with local complications and organ failure, presenting complex treatment challenges, significant economic burden, and poor prognosis, with mortality rates reaching up to 30% (Hamesch et al., 2025). Therefore, early assessment of AP severity to adjust treatment strategies and implement interventions is crucial for improving outcomes (Szatmary et al., 2022).

There are a number of clinical grading systems currently available for use in predicting disease severity, including the Ranson score, the Acute Physiology and Chronic Health Evaluation II (APACHE II) score, and the Bedside Index of Severity of Acute Pancreatitis (BISAP) (Zerem et al., 2023). C-reactive protein (CRP) and the CRP/lymphocyte ratio (CLR) are also employed to assess the severity of acute pancreatitis (Khan, 2020; Chen et al., 2024a). Limitations of these scoring systems include their requirement for multiple parameters, some of which are not routinely measured variables, necessitating repeated measurements and restricting their practical application. However, Clinical conditions are highly complex, and a single indicator cannot fully reflect their nuances. Machine learning (ML), with its robust computational and learning capabilities, has been widely applied in healthcare.

Research indicates that ML has achieved significant accomplishments in the medical field, encompassing areas such as disease biomarker and complication prediction, diagnosis, and survival analysis (Zhou et al., 2024; Bai et al., 2025; Jiang et al., 2025; Zhang et al., 2025; Zhao et al., 2025). ML also plays a crucial role in predicting the severity of AP. Rahul Thapa’s team constructed three models—logistic regression (LR), neural networks, and Extreme Gradient Boosting (XGBoost) — by comparing them with the Harmless Acute Pancreatitis Score (HAPS) and BISAP. They ultimately concluded that XGBoost demonstrated superior capability in identifying AP risk stratification (Thapa et al., 2022). Ma Yuhu et al. based on clinical and CT imaging features, developed a ML modelling used to predict the severity of gallstone-associated pancreatitis (GSP) (Ma et al., 2024). Kailai Xiang et al. developed a multi-view model using deep learning (DL) algorithms to predict AP severity by integrating patient demographics, relevant laboratory indicators, and CT imaging data. Results demonstrated superiority of the multi-view model over single-view approaches (Xiang and Shang, 2025). These studies indicate significant potential for ML techniques in predicting AP severity.

ML, with its capacity for deep analysis of high-dimensional data, has established novel pathways for SAP early warning systems. Laboratory tests conducted upon admission are readily obtainable at an early stage without imposing any additional burden on patients, providing robust support for the clinical implementation of predictive models. Consequently, this study aims to construct and validate a real-time interactive SAP risk prediction model using laboratory indicators as the sole data source. Concurrently, an online tool will be developed to enable early warning, thereby providing decision support for precise triage and timely intervention.

## 2 Methods

### 2.1 Data

**Figure 1:**
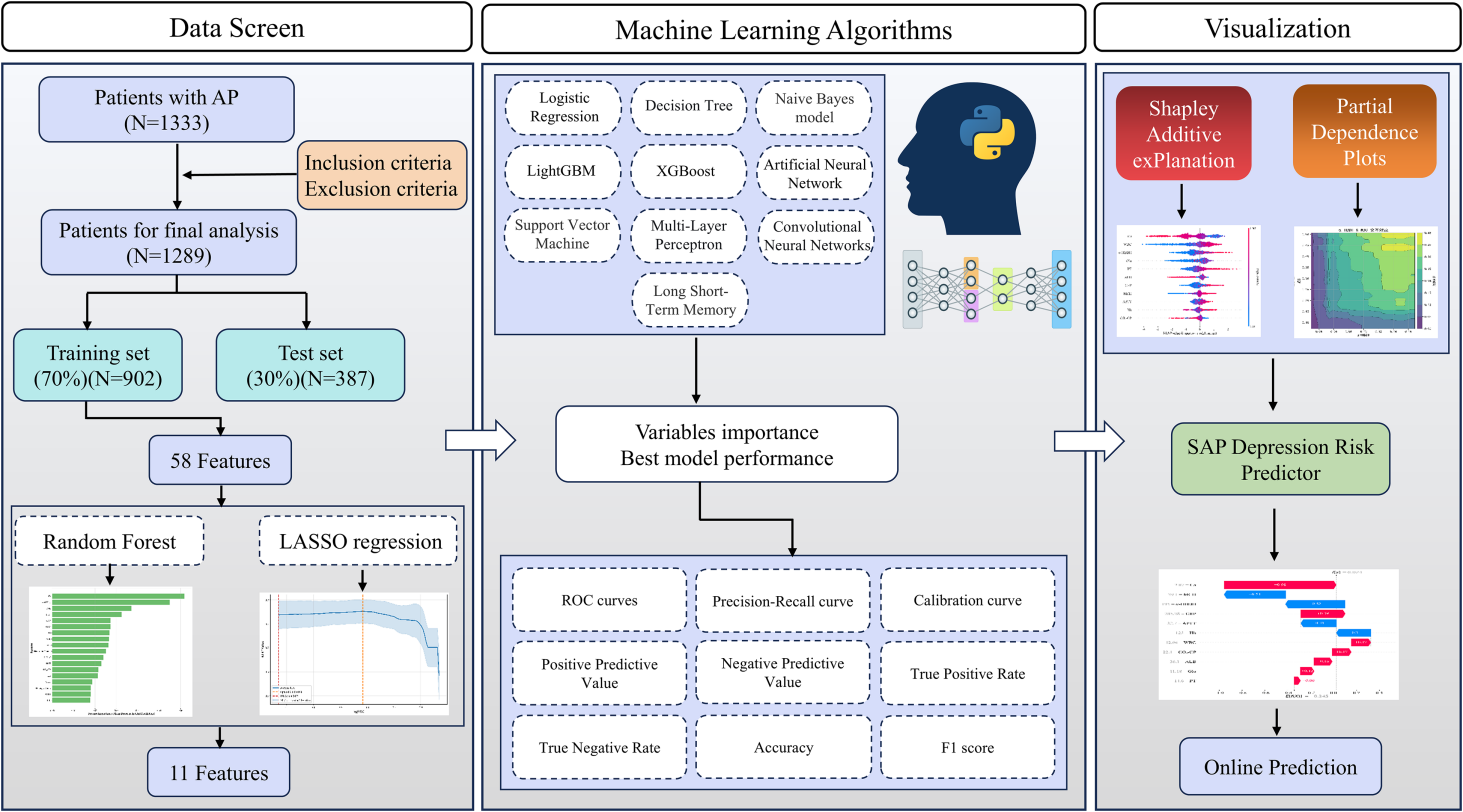
Overall flowchart of the study.

This is a retrospective observational study. All patients diagnosed with AP admitted to the Second Affiliated Hospital of Guilin Medical University between January 2016 and May 2025. It compiled data on the patients’ routine examination indexes within 24 hours of admission. The human samples used in this study followed the principles of the Declaration of Helsinki, were approved by the Ethics Committee of the Second Affiliated Hospital of Guilin Medical University (NO. ZLXM-2024013), and informed consent was obtained from the patients and/or their legal guardians. Following the inclusion and exclusion criteria, 1,289 individuals were ultimately enrolled. The sample selection process is detailed in **Figure 1**. SAP is based on the 2012 Atlanta Classification criteria (Banks et al., 2013). The inclusion criteria were (1) meeting the diagnostic criteria for AP; (2) age ≥ 18 years; and (3) admission to the hospital with complete indicators of the required tests. The exclusion criteria were (1) age < 18 years; (2) nursing or pregnant women; (3) patients with various malignant tumors; (4) patients with coagulation system disorders; (5) patients with chronic pancreatitis; (6) other acute abdominal diseases;(7) severe hepatic or renal dysfunction; (8) patients with a large number of missing test results after admission.

### 2.2 Data processing and feature selection

The patient’s relevant data primarily encompass the following examinations: routine blood tests, electrolytes, amylase (AMY), liver function, coagulation function, renal function, cardiac enzymes, lipid profile, CRP, and other standard indicators. Features with missing values exceeding 20% were deleted, while those with less than 20% missing values were imputed. This study employed the K Nearest Neighbor algorithm (KNN) algorithm for imputation. To address the issue of class imbalance, this study employed the Synthetic Minority Over-sampling Technique (SMOTE).

The dataset was divided into training set (70%) and test set (30%). Within the training set, we employed Random Forest (RF) and Least Absolute Shrinkage and Selection Operator (LASSO) regression analysis to identify features for the SAP. The optimal λ parameter for the LASSO regression was determined with 10-fold co-validation. Feature selection aims to obtain a robust set of predictors. Random forests were employed for this initial screening due to their low assumptions about data distribution and effective handling of nonlinear relationships, leveraging their built-in feature importance evaluation based on tree models. Subsequently, we used this feature subset to fairly compare the predictive performance of multiple machine learning algorithms. The final model was selected based on optimal overall performance. All subsequent analyses and interpretations were based on this model. We utilised the variance inflation factor (VIF) to detect potential covariance among features, interpreting VIF ≥ 10 as an indicator of multicollinearity. These features were employed to construct models including LR, Decision Trees (DT), Naive Bayes (NB), Support Vector Machines (SVM), Multi-Layer Perceptrons (MLP), Light Gradient Boosting Machine (LightGBM), XGBoost, Artificial Neural Networks (ANN), Convolutional Neural Networks (CNN), and Long Short-Term Memory Networks (LSTM) to predict SAP patients.

It should be noted that RF in this study is primarily used to assess feature importance (based on average Gini impurity reduction) to assist feature selection. It is not employed as an independent predictive model for cross-validation performance testing. We did not set an absolute Gini index threshold for RF but instead adopted a consensus strategy. We intersected the features selected by LASSO regression with the top-ranked features in the RF importance rankings to obtain a more robust feature subset. This intersection was then tested for variance inflation factors (VIF < 10) to exclude multicollinearity, ultimately serving as the basis for constructing and comparing all subsequent prediction models.

To improve predictive performance, we performed systematic hyperparameter optimization for the seven traditional machine learning models used in the study. A random search strategy with 5-fold cross-validation was employed to tune key hyperparameters within reasonable ranges for each model. The search spaces were defined as: regularization strength C (0.01-100) and penalty type (L1, L2) for LR; maximum depth (3-20) and minimum samples split (2-20) for DT; smoothing parameter alpha (0.1-2.0) for NB; kernel function (linear, RBF), penalty coefficient C (0.1-10), and kernel coefficient gamma (0.001-1) for SVM; hidden layer neurons (50-200) and learning rate (0.0001-0.01) for MLP; number of trees (50-500), learning rate (0.01-0.3), and maximum depth (3-15) for both LightGBM and XGBoost. The parameter set yielding the highest AUC on the validation set was selected to build all final models.

To assess the predictive performance of the models under evaluation, a comprehensive set of metrics was computed. This suite included both categorical performance measures and graphical evaluation tools: specifically, positive predictive value (PPV), negative predictive value (NPV), true positive rate (TPR), true negative rate (TNR), accuracy (ACC), and F1 score were calculated alongside three key graphical metrics — receiver operating characteristic (ROC) curves, precision-recall curves (PRC), and calibration curves. Together, these metrics collectively provide a robust framework for quantifying and visualizing the models’ ability to make accurate predictions.

### 2.3 Model explainability

After selecting the highest-performing model, Shapley Additive Explanations (SHAP) and partial dependence plots (PDP) were employed to analyse the impact of variables on SAP risk. SHAP is an explainable artificial intelligence tool that identifies the most significant factors and their contribution to predictions (Cao et al., 2025). Employing non-linear regression methods, PDP serve as a graphical tool to aid in interpreting the relationship between a model’s predictions and one or more independent variables (Zhang et al., 2025).

### 2.4 Statistical analysis

Patient data were statistically analyzed through the use of SPSS software, specifically version 25.0. Data exhibiting a normal distribution were presented in the format of mean ± standard deviation, whereas skewed data were documented as median (interquartile range). Categorical variables were described by means of percentage values (%). The full process of model creation was executed using Python, with version 3.8.5 as the operating environment. All datasets and source code related to this research are publicly available on GitHub at the following address: https://github.com/longshike/Predicting-acute-pancreatitis-severity-with-multi-machine-learning-models.

## 3 Results

### 3.1 Patient characteristics

1333 cases in our cohort met the AP criteria, with 1289 ultimately included in the study. Among these, 204 patients had SAP and 1085 had mild acute pancreatitis (MAP). The training set comprised 902 patients, with the leftover 387 individuals assigned to the validation set. The observed indicators for patients diagnosed with AP are detailed in **Table 1**; **Supplementary Table 1**.

**Table 1:** Baseline clinical characteristics of patients.

Variable	MAP (n = 1085)	SAP (n = 204)	P value
Sex			0.23
Male	712 (65.6%)	125 (61.3%)	
Female	373 (34.4%)	79 (38.7%)	
White Blood Cell Count (WBC) (10^9^/L)	11.70 (8.80, 15.08)	15.51 ± 4.99	0.00
Red Blood Cell Count (RBC) (10^12^/L)	4.69 (4.23, 5.13)	4.78 ± 0.99	0.21
Hemoglobin (Hb) (g/L)	140.60 (126, 156)	147.11 ± 31.62	0.00
Platelet Count (PLT) (10^9^/L)	230 (187, 283)	228.50 (184.50, 284.75)	0.78
Neutrophils Percentage (NEU%) (%)	80.80 (72.60, 86.90)	86.55 (80.33, 90.18)	0.00
Neutrophils Absolute Count (NEU#) (10^9^/L)	9.36 (6.58, 12.59)	12.93 (9.58, 16.32)	0.00
Lymphocytes Percentage (LYM%) (%)	11.70 (7.25, 18.45)	7.40 (4.70, 11.58)	0.00
Lymphocytes Absolute Count (LYM#) (10^9^/L)	1.32 (0.89, 1.89)	1.15 (0.77, 1.62)	0.00
Monocytes Percentage (MON%) (%)	5.90 (4.70, 7.60)	5.20 (4.03, 6.80)	0.00
Monocytes Absolute Count (MON#) (10^9^/L)	0.69 (0.50, 0.92)	0.79 (0.51, 1.13)	0.00
Eosinophils Percentage (EOS%) (%)	0.40 (0.10, 1.25)	0.10 (0, 0.40)	0.00
Eosinophils Absolute Count (EOS#) (10^9^/L)	0.05 (0.01, 0.13)	0.02 (0, 0.06)	0.00
Basophil Percentage (BAS%) %	0.20 (0.10, 0.40)	0.20 (0.10, 0.30)	0.00
Basophil Absolute Count (BAS#) (10^9^/L)	0.03 (0.02, 0.04)	0.03 (0.02, 0.04)	0.84
Hematocrit (HCT) (%)	41.80 (38.10, 44.90)	42.1 ± 8.07	0.25
Mean Corpuscular Volume (MCV) (fL)	89.70 (85.60, 93.35)	89.80 (86.43, 93.38)	0.41
Mean Corpuscular Hemoglobin (MCH) (pg)	30.50 (29, 31.80)	30.70 (29.43, 32.70)	0.00
Mean Corpuscular Hemoglobin Concentration (MCHC) (g/L)	336 (326, 347)	338 (326, 354.75)	0.01
Red Cell Distribution Width - Coefficient of Variation (RDW-CV) (%)	13 (12, 14)	13 (13, 14)	0.00
Red Cell Distribution Width - Standard Deviation (RDW-SD) (fL)	41.90 (39.30, 44.80)	42.80 (40.70, 45.60)	0.00
Plateletcrit (PCT) (%)	0.23 (0.19, 0.28)	0.24 (0.20, 0.30)	0.10
Mean Platelet Volume (MPV) (fL)	10.10 (9.40, 10.80)	10.30 (9.70, 11)	0.00
Platelet Distribution Width (PDW) (%)	11.50 (10.10, 13)	12 (10.53, 13.50)	0.00
Platelet Large Cell Ratio (P-LCR) (%)	25.60 (20.60, 31.50)	27.45 (22.73, 33.38)	0.00
C-Reactive Protein (CRP) (mg/L)	20.36 (4.58, 68.55)	92.27 (22.38, 175.98)	0.00
Total Protein (TP) (g/L)	73.50 (68.81, 77.70)	70.85 (64.80, 76.48)	0.00
Albumin (ALB) (g/L)	41.90 (38, 44.90)	37.75 (31.40, 42.58)	0.00
Globulin (GLOB) (g/L)	31.60 (28.30, 35.80)	33.50 (29.20, 36.70)	0.00
Albumin/Globulin Ratio (A/G)	1.31 (1.10, 1.50)	1.13 (0.94, 1.34)	0.00
Total Bilirubin (TBIL) (μmol/L)	12.80 (8.20, 21.40)	14.85 (9.28, 29.28)	0.00
Indirect Bilirubin (IBIL) (μmol/L)	6.60 (4.50, 10.10)	7.05 (4.20, 11.10)	0.00
Direct Bilirubin (DBIL) (μmol/L)	5.30 (3, 10.45)	7.25 (4.13, 15.28)	0.00
Alanine Aminotransferase (ALT) (U/L)	29 (16.35, 66.25)	33.20 (15.63, 74.78)	0.87
Aspartate Aminotransferase (AST) (U/L)	29.10 (19.45, 70.30)	36.70 (22.10, 71.15)	0.00
AST/ALT Ratio	1.12 (0.78, 1.68)	1.33 (0.87, 2.06)	0.00
Gamma-Glutamyl Transferase (GGT) (U/L)	88 (46, 187)	110 (58, 189.55)	0.07
Alkaline Phosphatase (ALP) (U/L)	80.50 (66, 103.10)	83.04 (65.81, 115.43)	0.29
Creatinine (Cr) (μmol/L)	72 (61, 85)	77 (63, 108.75)	0.00
Urea (mmol/L)	4.64 (3.60, 6.10)	5.30 (3.90, 8.08)	0.00
Carbon Dioxide Combining Power (CO_2_CP) (mmol/L)	23.40 (21.20, 25.40)	21.05 (17.50, 24.08)	0.00
Creatine Kinase (CK) (U/L)	94 (63, 151)	95.50 (66, 183)	0.15
Creatine Kinase-MB (CK-MB) (ng/ml)	14.60 (10.70, 20.80)	17.40 (11.83, 27.75)	0.00
Lactate Dehydrogenase (LDH) (U/L)	228 (185, 298)	327 (236.25, 535.75)	0.00
Alpha-Hydroxybutyrate Dehydrogenase (α-HBDH) (U/L)	163 (134, 209)	244.20 (174.14, 356)	0.00
Amylase (AMY) (U/L)	306 (113.95, 955)	585 (138, 1160.70)	0.00
Potassium (K) (mmol/L)	3.84 (3.60, 4.10)	3.84 (3.57, 4.22)	0.44
Chloride (Cl) (mmol/L)	101.60 (98.30, 104.10)	100.90 (96.43, 103.75)	0.03
Sodium (Na) (mmol/L)	139 (136, 141)	137.45 (133.90, 140.23)	0.00
Calcium (Ca) (mmol/L)	2.25 (2.14, 2.33)	2.06 (1.89, 2.20)	0.00
Triglycerides (TG) (mmol/L)	3.05 (1.13, 8.47)	3.47 (1.30, 10.81)	0.07
Cholesterol (CHOL) (mmol/L)	5 (4.02, 6.24)	4.82 (3.84, 6.96)	0.82
Low-Density Lipoprotein Cholesterol (LDL-C) (mmol/L)	2.30 (1.51, 2.91)	1.81 (0.87, 2.71)	0.00
Prothrombin Time (PT) (S)	12.90 (12.20, 13.70)	13.90 (12.80, 15.28)	0.46
International Normalized Ratio (INR)	1.03 (0.97, 1.10)	1.12 (1.01, 1.26)	0.00
Activated Partial Thromboplastin Time (APTT) (S)	33.20 (30, 36.90)	35.65 (32.23, 41.30)	0.00
Fibrinogen (FIB) (g/L)	3.70 (3, 4.84)	4.62 (3.51, 6.50)	0.00
Thrombin Time (TT) (S)	16.50 (15.50, 17.88)	16.60 (15.40, 18.40)	0.00
Glucose (Glu) (mmol/L)	7.56 (6.17, 9.57)	9.43 (7.57, 13.05)	0.00

### 3.2 Feature selection

**Figure 2:**
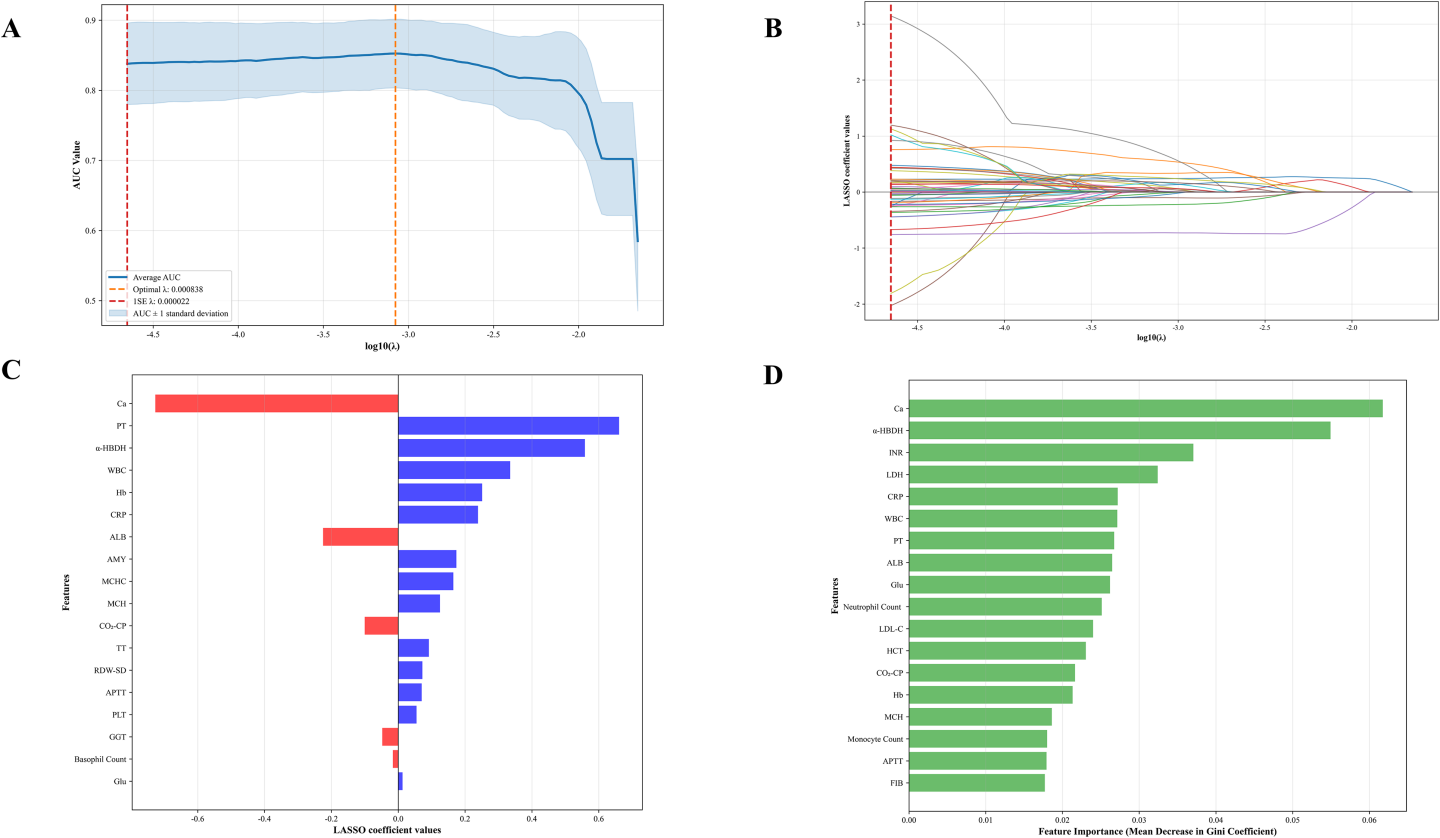
Feature engineering. **(A)** Selection of optimal parameters (λ) using LASSO regression and 10-fold cross-validation. **(B)** The LASSO coefficients for each feature vary with different parameters (λ). **(C)** Coefficient profile of the characteristics included in the LASSO for predicting the ARF in the λ at 1 standard error. **(D)** The relative influence of various variables on the construction of trees in RF.

LASSO regression and RF analyses were conducted on the 58 variables within the training set. LASSO method determined that λ = 1 standard error (0.000838) represented the best penalty parameter, leading to the final selection of 18 variables (**Figure 2C**). RF similarly selected 18 variables based on mean reduction in Gini coefficient (**Figure 2D**). The variables selected by LASSO intersected with those from RF. Considering covariance and mean reduction in Gini value, the remaining variables — WBC, α-HBDH, APTT, PT, Ca, CO_2_-CP, Hb, CRP, MCH, Glu, and ALB — were used to construct the clinical prediction model (**Table 2**).

**Table 2:** Results of the VIF test.

Variable	VIF	Variable	VIF
Hb	1.861758	α-HBDH	1.309851
ALB	1.779307	APTT	1.294677
Ca	1.695566	CO_2_-CP	1.284710
CRP	1.412527	Glu	1.229668
MCH	1.399220	WBC	1.202197
PT	1.349832		

### 3.3 Model performance

**Figure 3:**
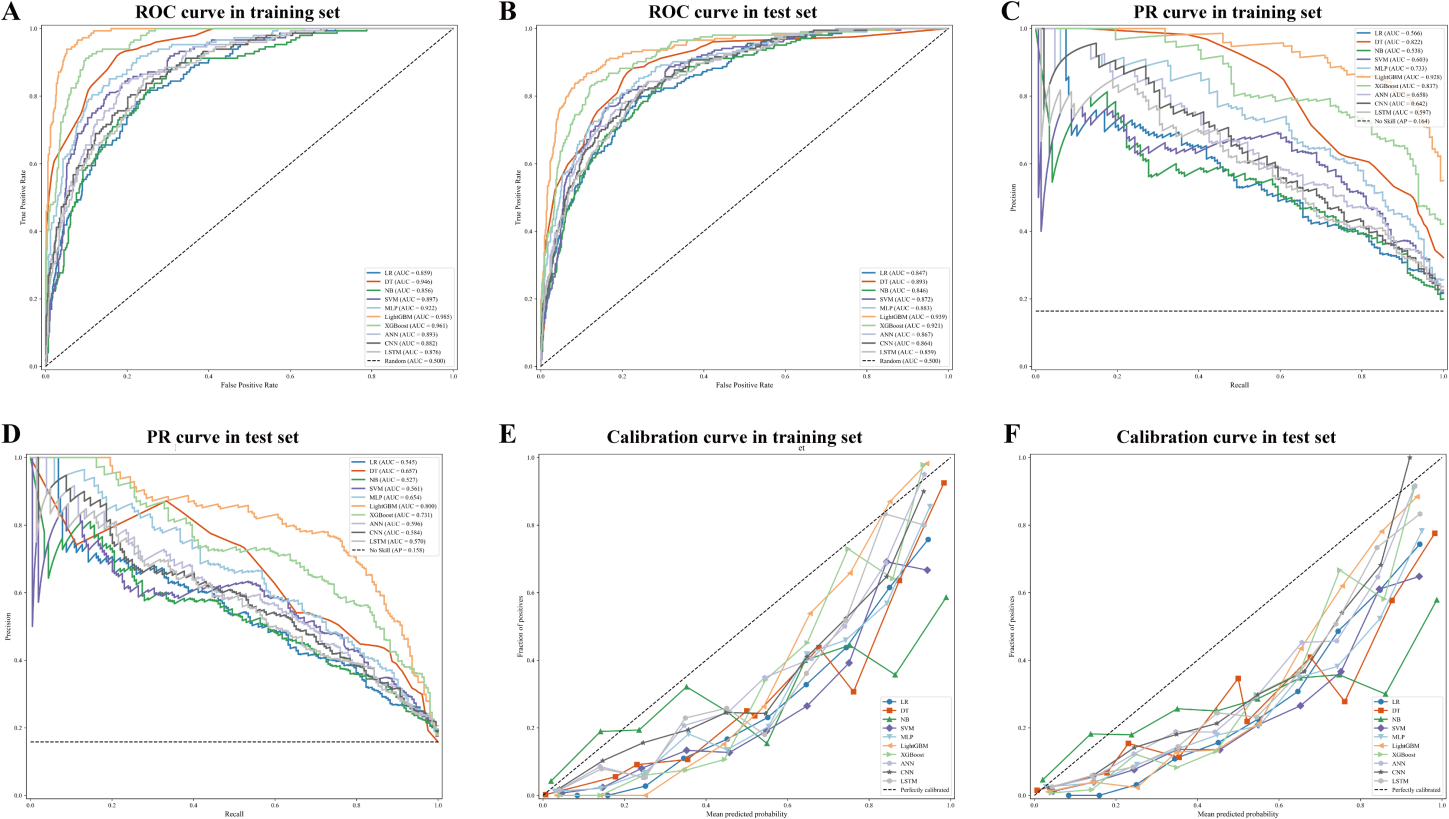
Performance and comparison of ten distinct predictive models. **(A)** ROC curve for the training set; **(B)** ROC curve for the validation set; **(C)** PRC for the training set; **(D)** PRC for the validation set; **(E)** Calibration curve for the training set; **(F)** Calibration curve for the validation set.

On the training and validation sets, LightGBM achieved an AUC (95% CI) of 0.9852 (0.9792-0.9907) and 0.9386 (0.9188-0.9560), outperforming the LR, DT, NB, SVM, MLP, XGBoost, ANN, CNN, and SLTM models (**Figures 3A, B**). Comparing model performance in the PRC, the LightGBM model achieved the best stability compared to the other nine models (**Figures 3C, D**). Furthermore, calibration plots demonstrated that the LightGBM model also outperformed other models, further supporting the reliability of the LightGBM model (**Figures 3E, F**), **Table 3**.

**Table 3:** Detailed performance metrics of various machine learning models for predicting the severity risk in AP patients, across the training and validation sets.

Group	Model	ROC_AUC (95%CI)	Accuracy	Precision	Recall	F1	TPR	TNR	PPV	NPV	PRC_AUC (95%CI)
Training set	LR	0.8590 (0.8294-0.8850)	0.7949	0.4257	0.7162	0.5340	0.7162	0.8103	0.4257	0.9357	0.5656 (0.4845-0.6404)
	DT	0.9460 (0.9290-0.9613)	0.8537	0.5328	0.8784	0.6633	0.8784	0.8488	0.5328	0.9726	0.8215 (0.7672-0.8685)
	NB	0.8562 (0.8241-0.8840)	0.8381	0.5057	0.6014	0.5494	0.6014	0.8846	0.5057	0.9187	0.5380 (0.4550-0.6201)
	SVM	0.8971 (0.8702-0.9185)	0.8126	0.4610	0.8378	0.5947	0.8378	0.8077	0.4610	0.9621	0.6032 (0.5192-0.6864)
	MLP	0.9222 (0.8998-0.9416)	0.8481	0.5234	0.8311	0.6423	0.8311	0.8515	0.5234	0.9625	0.7331 (0.6626-0.7970)
	LightGBM	0.9852 (0.9792-0.9907)	0.9390	0.7514	0.9392	0.8348	0.9392	0.9390	0.7514	0.9874	0.9279 (0.8956-0.9556)
	XGBoost	0.9613 (0.9494-0.9725)	0.9047	0.6505	0.9054	0.7571	0.9054	0.9045	0.6505	0.9799	0.8366 (0.7840-0.8830)
	ANN	0.8928 (0.8658-0.9152)	0.8514	0.5365	0.6959	0.6059	0.6959	0.8820	0.5365	0.9366	0.6581 (0.5846-0.7296)
	CNN	0.8816 (0.8520-0.9060)	0.8492	0.5337	0.6419	0.5828	0.6419	0.8899	0.5337	0.9268	0.6419 (0.5614-0.7140)
	LSTM	0.8757 (0.8459-0.8998)	0.8126	0.4533	0.6892	0.5469	0.6892	0.8369	0.4533	0.9321	0.5974 (0.5165-0.6766)
Test set	LR	0.8470 (0.8199-0.8721)	0.7967	0.4157	0.7010	0.5219	0.7010	0.8147	0.4157	0.9354	0.5451 (0.4739-0.6134)
	DT	0.8930 (0.8694-0.9159)	0.8123	0.4484	0.8088	0.5769	0.8088	0.8129	0.4484	0.9577	0.6574 (0.5901-0.7248)
	NB	0.8463 (0.8186-0.8718)	0.8363	0.4861	0.5980	0.5363	0.5980	0.8811	0.4861	0.9210	0.5274 (0.4533-0.6014)
	SVM	0.8721 (0.8461-0.8965)	0.8006	0.4297	0.7941	0.5577	0.7941	0.8018	0.4297	0.9539	0.5608 (0.4854-0.6372)
	MLP	0.8833 (0.8574-0.9082)	0.8270	0.4711	0.7598	0.5816	0.7598	0.8396	0.4711	0.9490	0.6542 (0.5861-0.7138)
	LightGBM	0.9386 (0.9188-0.9560)	0.8968	0.6310	0.8382	0.7200	0.8382	0.9078	0.6310	0.9676	0.8002 (0.7485-0.8469)
	XGBoost	0.9212 (0.9014-0.9393)	0.8704	0.5619	0.8235	0.6680	0.8235	0.8793	0.5619	0.9636	0.7307 (0.6721-0.7857)
	ANN	0.8672 (0.8420-0.8918)	0.8479	0.5154	0.6569	0.5776	0.6569	0.8839	0.5154	0.9320	0.5961 (0.5236-0.6635)
	CNN	0.8641 (0.8385-0.8886)	0.8379	0.4908	0.6569	0.5618	0.6569	0.8719	0.4908	0.9311	0.5843 (0.5119-0.6542)
	LSTM	0.8587 (0.8329-0.8836)	0.8130	0.4405	0.6716	0.5320	0.6716	0.8396	0.4405	0.9315	0.5704 (0.4954-0.6406)

### 3.4 Model interpretability and application

**Figure 4:**
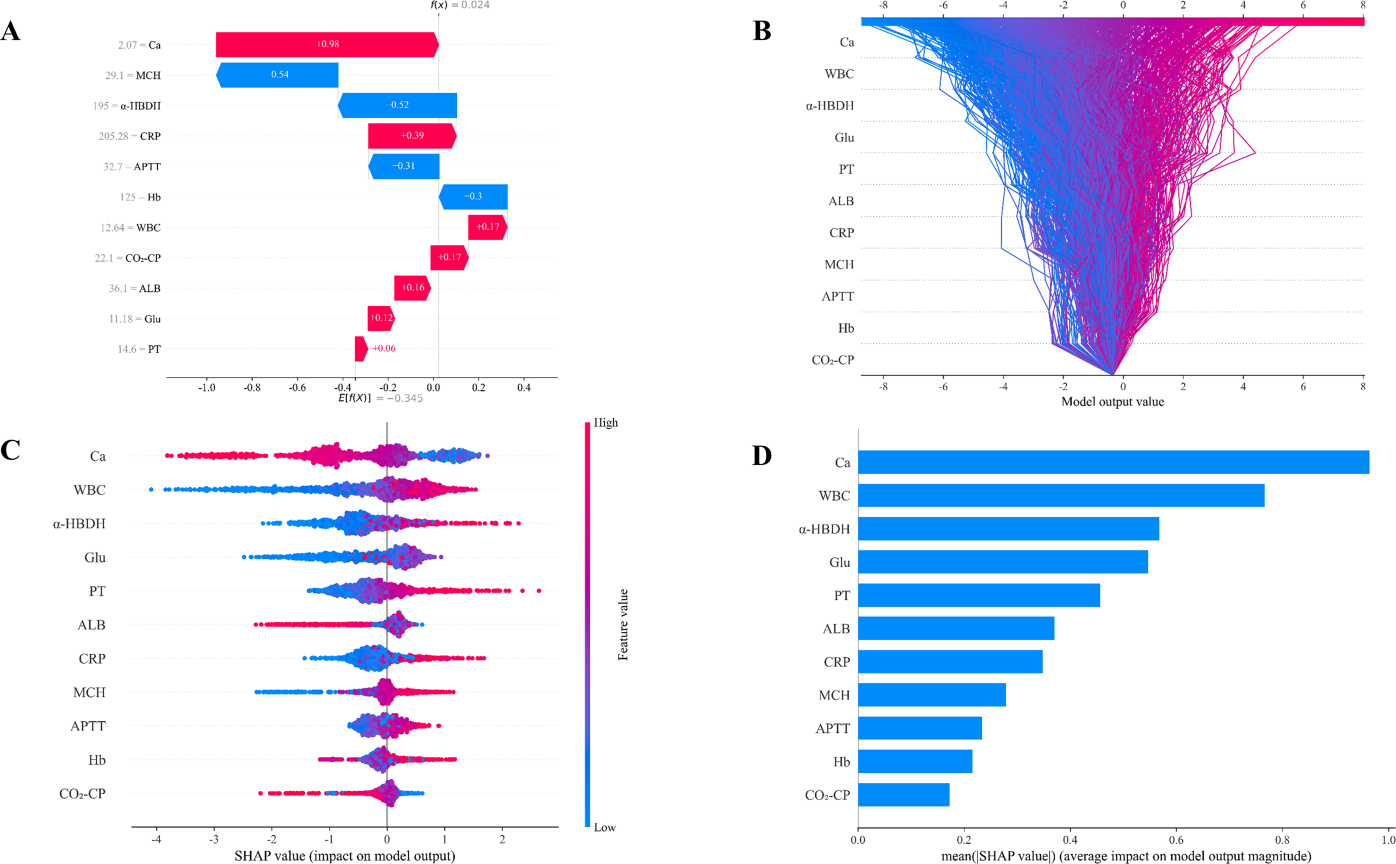
SHAP analysis. **(A)** SHAP waterfall plot. **(B)** SHAP decision tree. **(C)** SHAP swarm plot. **(D)** SHAP bar chart.

SHAP visualizations corresponding to the LightGBM model serve to emphasize the primary features linked to the risk of SAP. In **Figure 4A**, red bars indicate the variable’s contribution to individual SAP progression, while blue bars represent its inhibitory effect. For instance, a patient with serum Ca of 2.07 mmol/L, CRP of 205.28 mg/L, and WBC of 12.64 × 10^9^/L exhibits increased SAP risk. **Figure 4B** depicts the decision tree for each participant, whilst **Figure 4C** illustrates the extent of each feature’s influence on SAP. Every data point corresponds to a feature’s Shapley value for a specific instance, and color is used to indicate the feature’s magnitude — with red signifying high values and blue representing low ones. Features are ordered based on their significance, and their placement along the horizontal axis shows how much they contribute to the model’s results. Greater SHAP values are associated with a higher likelihood of SAP. **Figure 4D** reveals the 11 variables most predictive of SAP severity based on importance ranking. The results indicate that Ca exhibits a negative correlation with predicted SAP occurrence, whereas WBC, α-HBDH, and Glu demonstrate positive correlations with predicted SAP occurrence.

### 3.5 The influence of a single feature on predicting SAP

**Figure 5:**
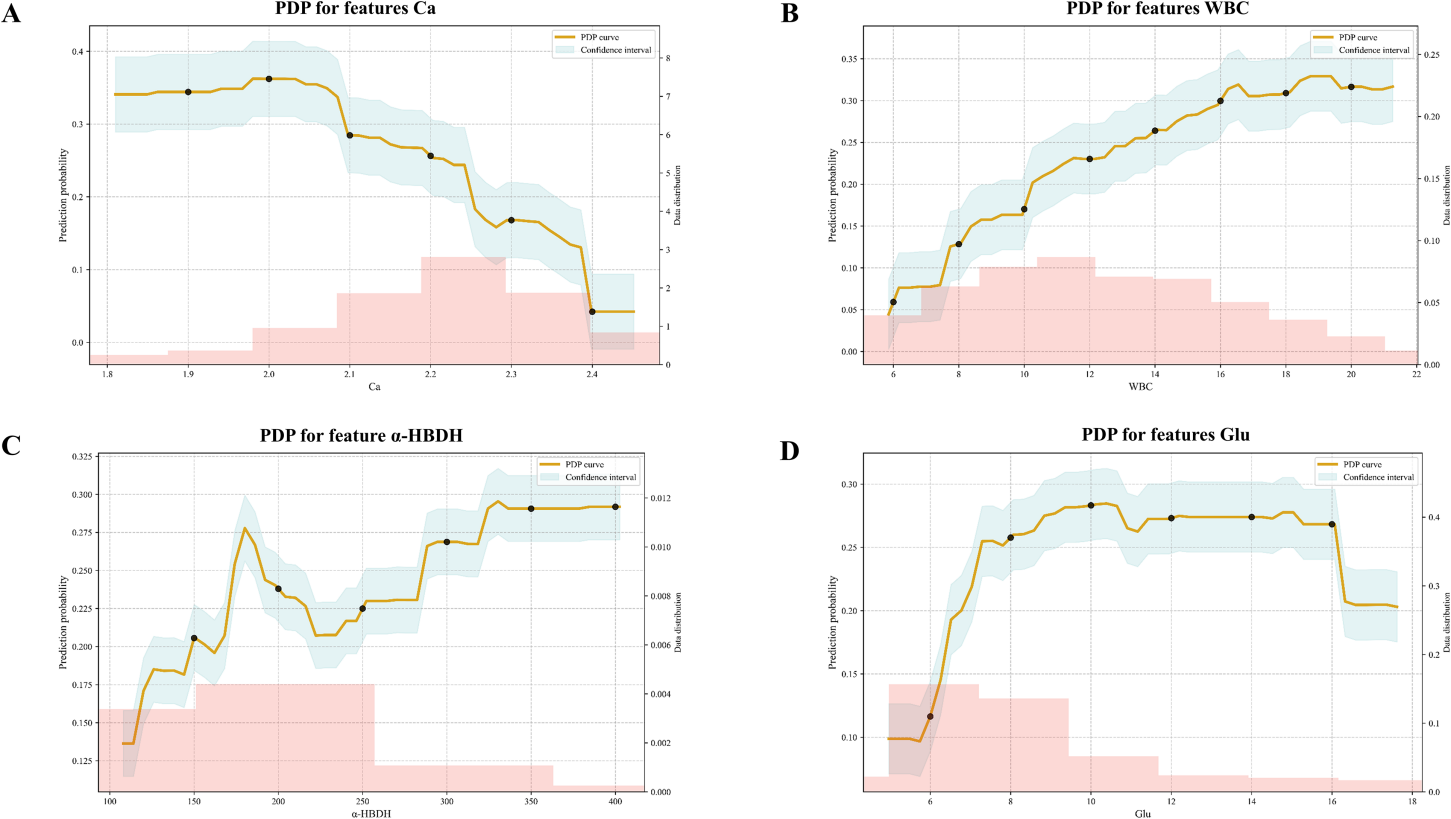
Relationship between features and SAP. The PDP illustrates the relationship between SAP and **(A)** Ca, **(B)** WBC, **(C)** α-HBDH, and **(D)** Glu. The horizontal axis displays each feature’s logarithmically transformed value, with the vertical axis showing changes in the predicted risk of SAP. Confidence intervals are represented by shaded areas, and the pink histograms below each graph illustrate how data points are distributed across different feature values.

The application of PDP enables a more comprehensive understanding of how individual features influence SAP risk prediction. **Figure 5** illustrates the relationships among four key features (Ca, WBC, α-HBDH, Glu) within the LightGBM model and their impact on SAP risk. PDP analysis indicates that lower Ca, higher WBC, α-HBDH, and Glu levels correlate with increased SAP risk, reinforcing conclusions drawn from the preceding SHAP analysis. For instance, **Figure 5A** shows that when log-transformed Ca levels exceed 2.0, SAP risk diminishes. **Figure 5B** shows an increase with higher WBC values; **Figure 5C** exhibits variation with α-HBDH; **Figure 5D** displays a sharp rise followed by plateau and decline for Glu. PDPs for the remaining seven variables incorporated into the constructed machine learning model are presented in **Supplementary Figure 1**.

### 3.6 Key features synergising with SAP

**Figure 6:**
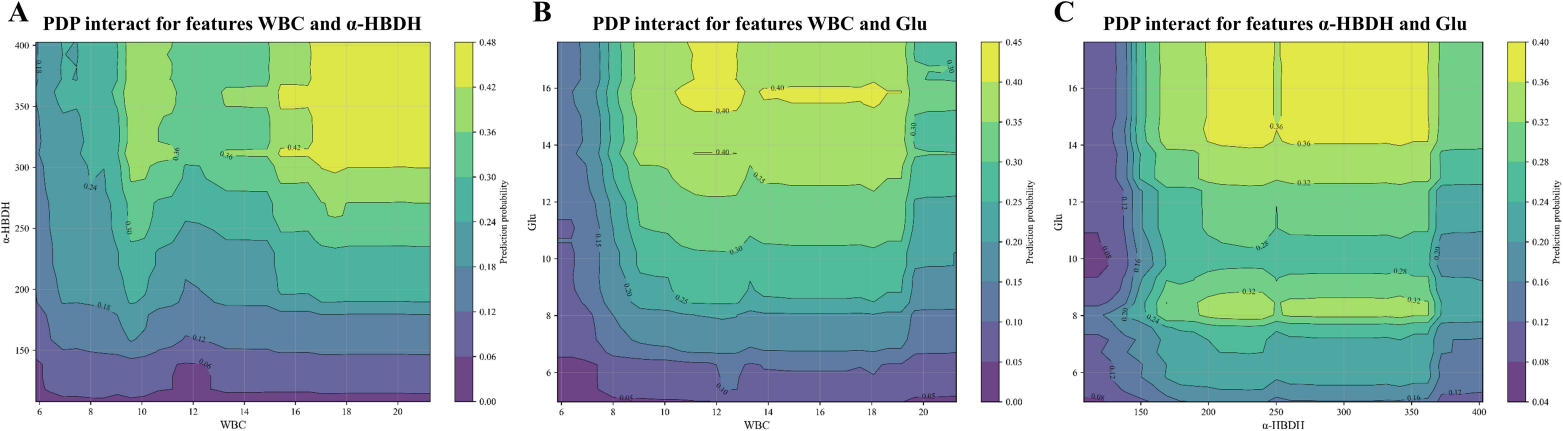
Synergistic effects of key features on SAP. The PDP illustrates the synergistic effects of **(A)** WBC and α-HBDH, **(B)** WBC and Glu, and **(C)** α-HBDH and Glu on SAP. The horizontal axis and vertical axis stand for the logarithmically transformed values of the two features, where each grid cell corresponds to a 10% range of the feature’s level. The contour graph illustrates how these two features affect the predicted outcomes of SAP.

PDP further reveals how the two variables interact when predicting SAP. As Ca has been established as an independent clinical predictor of SAP (Qi et al., 2024), it was not compared for synergistic effects. **Figure 6A** indicates that elevated WBC and elevated α-HBDH increase SAP risk; **Figures 6B, C** reveal a noteworthy finding: when Glu is elevated, the predicted risk of SAP is determined by WBC and α-HBDH, yet the relationship between these two and Glu is complex.

We built a deployable web platform using Streamlit to visualize and apply predictive models (access link: https://online-predictor-for-acute-pancreatitis-severity-appappgit-yw5.streamlit.app/). Designed as an online tool specifically for assessing the risk of SAP occurrence, users can easily obtain the desired predictive results by directly inputting clinical feature data into the designated text boxes on the webpage.

## 4 Discussion

Accurate prediction of AP severity is crucial for determining patient treatment strategies, particularly for those at risk of developing SAP. Individuals diagnosed with SAP demand intensive care and immediate medical actions, such as quick fluid replacement, enteral feeding, and pain control — all of which call for hospital-based resources (Zhou et al., 2022; Luo et al., 2023). Furthermore, surgical intervention may be required for patients with necrotising SAP (Zhou et al., 2022). Consequently, identifying SAP patients early is crucial to ensuring they have timely access to necessary treatment and care, thereby contributing to improved therapeutic outcomes and patient survival rates (Tan et al., 2024).

Through LASSO regression and RF, we determined 11 key feature variables. As shown in **Figure 4**, lower Ca, CO_2_-CP, and higher WBC, α-HBDH, Glu, PT, CRP, MCH, APTT, and Hb increased the risk of predicting SAP. Conversely, higher ALB indicated a concurrent reduction in the risk of predicting SAP. The relationship between these factors and AP has been extensively studied in previous research. Serum Ca serves as an independent clinical predictor of SAP (Qi et al., 2024), and low Ca levels are predictive of intensive care unit admission or mortality (Yan et al., 2024). Early serum α-HBDH levels in AP patients correlate closely with poor prognosis and may serve as a potential early biomarker for predicting AP severity (Xiao et al., 2020). Yuxin Liu et al. observed significantly elevated Glu levels in the SAP group compared to the MAP group (Liu et al., 2025), Glu constitutes an independent risk factor for systemic inflammatory response syndrome (SIRS) in AP patients (Zou et al., 2025) and correlates positively with SAP (Wang et al., 2025). Our results indicate that CRP and WBC levels positively correlate with AP severity, consistent with findings by Qi et al (Qi et al., 2024). WBC represents the most routinely available and readily accessible clinical inflammatory marker. Research indicates that WBC is a key predictor of postoperative pancreatic fistula in machine learning models developed for pancreatic surgery (Potievskiy et al., 2025). Although its specificity is limited (it is elevated in numerous infectious and non-infectious conditions), its degree of elevation in AP correlates closely with disease severity (Bedel et al., 2021; Li et al., 2024b; Huang et al., 2025). CRP remains the most commonly used and reliable inflammatory marker for monitoring AP disease progression and prognosis. Its peak levels and duration of elevation correlate directly with organ failure and mortality (He et al., 2024; Liţă Cofaru et al., 2025). Coagulation dysfunction plays a pivotal role in the pathogenesis of SAP (Gui et al., 2023). Compared with the non-SAP group, the PT was markedly elevated in the SAP group (Xu et al., 2023), and PT is a risk factor for SAP (Yang et al., 2024), PT increases with worsening AP and shows a positive correlation with AP severity (Peng et al., 2025). Prolonged APTT is a predictor of AP-related mortality and organ failure (Liu et al., 2019; Li et al., 2024a). Serum ALB constitutes an independent protective factor in AP patients (Lu et al., 2022), which is significantly negatively correlated with AP severity (Chen et al., 2024b; Zhang, 2025). Studies indicate (Komara et al., 2020; Nian et al., 2025), hat ALB levels decrease markedly during SAP or severe inflammation, serving as a definitive indicator of disease severity. The intense inflammatory response in early SAP causes substantial fluid leakage into the third space, leading to blood concentration (Komara et al., 2020). with relative increases in Hb and MCH concentrations reflecting early manifestations of severe effective circulating blood volume depletion. AP patients frequently develop metabolic disorders due to inflammatory responses, tissue necrosis, and infection, leading to acid-base imbalance. Changes in CO_2_-CP reflect the patient’s acid-base equilibrium status, aiding in assessing disease severity and guiding treatment (Seifter and Chang, 2017; Achanti and Szerlip, 2023).

The LightGBM model outperformed the other nine ML models in terms of AUC (95% CI), PPV, NPV, TPR, TNR, ACC, and F1 score on both the training and validation sets. On the PRC of the test and validation sets, LightGBM demonstrated robust performance compared to the other nine ML models, achieving AUC (95% CI) values of 0.8731 (0.8279-0.9152) and 0.7490 (0.6887-0.8050), respectively. Furthermore, when evaluating model calibration using calibration curves, LightGBM demonstrated strong calibration and predictive accuracy on both the test and validation sets. In summary, the LightGBM model demonstrated excellent generalisability and robustness, leading to its selection as the optimal predictive tool. This study selected LightGBM as the optimal model based on a multidimensional evaluation. Beyond achieving the highest AUC, it demonstrated the best area under the precision-recall curve, indicating greater robustness in identifying the minority class (SAP). Its calibration curves most closely approached the ideal diagonal line, reflecting more reliable prediction probabilities. Additionally, as a gradient boosting framework, it exhibits highly efficient computational performance, facilitating clinical deployment. Consequently, LightGBM holds a comprehensive advantage in discriminative power, stability, calibration accuracy, and efficiency. LightGBM has exhibited efficient computational performance and robust generalisation capabilities across numerous medical applications, such as predictive models for postoperative complications in gastric cancer and 28-day mortality prediction in elderly neurocritical care patients (Wang et al., 2024; Yuan et al., 2025).

The application of machine learning in medicine varies depending on the data and objectives. Traditional statistical models such as LR emphasize interpretability. Deep learning excels at handling high-dimensional data like images or sequences. Ensemble methods like gradient boosting machines typically demonstrate robustness in predicting structured tabular data. This study aims to develop a rapid early warning tool based on routine indicators, thus prioritizing a comparison between traditional and ensemble approaches. LightGBM, as an efficient gradient boosting implementation, excels in structured data prediction by balancing accuracy and speed, making it suitable for this research objective. Future work may explore more complex paradigms, such as multimodal data fusion, to further enhance performance.

Risk factors and scoring systems for assessing the severity of AP are complex, making prediction challenging even for experienced clinicians. ML approaches offer a superior method for building clinically applicable predictive models. This study incorporated data from within 24 hours before and after admission to general wards, identifying 11 key features for distinguishing AP severity. Among these, Ca, WBC, α-HBDH, and Glu emerged as the most critical indicators. These features are routine tests for outpatient, emergency, and/or inpatient care, being readily accessible and cost-effective, thereby enhancing the model’s practicality. Among ten ML models, LightGBM demonstrated superior predictive capability. To facilitate broader application, this study developed a user-friendly online prediction platform for the LightGBM model, accessible to clinicians worldwide at https://online-predictor-for-acute-pancreatitis-severity-appappgit-yw5.streamlit.app/. This research is not without its limitations, however. Firstly, this research was designed retrospectively, which to some extent limits the accurate assessment of the model’s predictive capability for SAP in prospective clinical settings. Furthermore, Although hyperparameter optimization via random search and cross-validation was conducted for the seven machine learning models, the chosen optimization strategy and parameter search spaces have inherent limitations. Future work could employ more efficient optimization algorithms and explore broader parameter spaces to further enhance model performance and ensure convergence to a global optimum.

Third, to prioritize model and feature interpretability, this study did not incorporate unsupervised dimensionality reduction methods such as Principal Component Analysis (PCA) into the feature selection process. Although PCA effectively handles high-dimensional data, the principal components it generates are linear combinations of original indicators, making their clinical significance less intuitive than individual indicators. This may impact the understanding and application of the predictive tool in clinical settings. Future research may explore the potential application of supervised dimensionality reduction or feature transformation methods while ensuring clinical interpretability.

Fourth, this study’s reliance on single-center data introduces potential selection bias, limiting the model’s external generalizability. We plan to establish a large-scale multicenter collaboration and carrying out rigorous, standardized external validation will be our top priorities and core objectives in our subsequent and extended investigations. These future efforts are aimed at systematically verifying, optimizing and strengthening the model’s external validity, real-world applicability and generalization ability across diverse populations and clinical settings.

Finally, Another limitation of this study is that the predictive performance of our machine learning model was not directly compared with established clinical scoring systems, such as the Ranson score, APACHE II score, or BISAP score. In the absence of such head-to-head comparative data, it is not possible within the scope of this study to quantitatively demonstrate whether the new model offers clear clinical superiority or incremental value over these classic tools. This comparison is crucial for evaluating the translational potential of the new model. Therefore, in subsequent extended research, we will prioritize conducting multicenter validation and systematically include traditional scores as benchmarks to clarify the relative efficacy and appropriate clinical application scenarios of our model.

## 5 Conclusions

Our research highlights the substantial predictive potential of Ca, WBC, α-HBDH, and Glu for SAP. We have created an online prediction platform tailored for clinical use, which allows healthcare providers to evaluate SAP risk quickly and efficiently. In turn, this supports the delivery of timely intervention and treatment for patients.

## Data Availability

All datasets and source code related to this research are publicly available on GitHub at the following address: https://github.com/longshike/Predicting-acutepancreatitis-severity-with-multi-machine-learning-models.

## References

[B1] Achanti A. Szerlip H. M. (2023). Acid-base disorders in the critically ill patient. Clin. J. Am. Soc. Nephrol. 18, 102–112. doi: 10.2215/cjn.04500422, PMID: 35998977 PMC10101555

[B2] Bai Q. Chen H. Gao Z. Li B. Liu S. Dong W. . (2025). Advanced prediction of heart failure risk in elderly diabetic and hypertensive patients using nine machine learning models and novel composite indices: insights from NHANES 2003-2016. Eur. J. Prev. Cardiol. 33, 53–63. doi: 10.1093/eurjpc/zwaf081, PMID: 40036490

[B3] Banks P. A. Bollen T. L. Dervenis C. Gooszen H. G. Johnson C. D. Sarr M. G. . (2013). Classification of acute pancreatitis--2012: revision of the Atlanta classification and definitions by international consensus. Gut 62, 102–111. doi: 10.1136/gutjnl-2012-302779, PMID: 23100216

[B4] Bedel C. Korkut M. Selvi F. (2021). New markers in predicting the severity of acute pancreatitis in the emergency department: Immature granulocyte count and percentage. J. Postgrad Med. 67, 7–11. doi: 10.4103/jpgm.JPGM_784_20, PMID: 33533745 PMC8098866

[B5] Cao J. Long S. Liu H. Chen F. Liang S. Fang H. . (2025). Constructing a prediction model for acute pancreatitis severity based on liquid neural network. Sci. Rep. 15, 16655. doi: 10.1038/s41598-025-01218-5, PMID: 40360617 PMC12075669

[B6] Chen X. Lin Z. Chen Y. Lin C. (2024a). C-reactive protein/lymphocyte ratio as a prognostic biomarker in acute pancreatitis: a cross-sectional study assessing disease severity. Int. J. Surg. 110, 3223–3229. doi: 10.1097/js9.0000000000001273, PMID: 38446844 PMC11175793

[B7] Chen X. Wang H. Geng P. Ling B. Ma A. Xu M. . (2024b). Serum Claudin-5 levels facilitate the early prediction of severe acute pancreatitis: a prospective observational study. Zhonghua Wei Zhong Bing Ji Jiu Yi Xue 36, 930–936. doi: 10.3760/cma.j.cn121430-20240318-00247, PMID: 39380513

[B8] Gui M. Zhao B. Huang J. Chen E. Qu H. Mao E. (2023). Pathogenesis and therapy of coagulation disorders in severe acute pancreatitis. J. Inflammation Res. 16, 57–67. doi: 10.2147/jir.s388216, PMID: 36636248 PMC9831125

[B9] Hamesch K. Hollenbach M. Guilabert L. Lahmer T. Koch A. (2025). Practical management of severe acute pancreatitis. Eur. J. Intern. Med. 133, 1–13. doi: 10.1016/j.ejim.2024.10.030, PMID: 39613703

[B10] He R. R. Yue G. L. Dong M. L. Wang J. Q. Cheng C. (2024). Sepsis biomarkers: advancements and clinical applications-A narrative review. Int. J. Mol. Sci. 25, 9010. doi: 10.3390/ijms25169010, PMID: 39201697 PMC11354379

[B11] Huang X. Xu J. Hu X. Yang J. Liu M. (2025). Development and validation of a visual prediction model for severe acute pancreatitis: a retrospective study. Front. Med. (Lausanne) 12. doi: 10.3389/fmed.2025.1564742, PMID: 40672827 PMC12263550

[B12] Jiang M. Wu X. P. Lin X. C. Li C. L. (2025). Explainable machine learning model for predicting acute pancreatitis mortality in the intensive care unit. BMC Gastroenterol. 25, 131. doi: 10.1186/s12876-025-03723-3, PMID: 40033198 PMC11877909

[B13] Khan J. A. (2020). “ Role of crp in monitoring of acute pancreatitis,” in Clinical significance of C-reactive protein (Singapore: Springer), 117–173.

[B14] Komara N. L. Paragomi P. Greer P. J. Wilson A. S. Breze C. Papachristou G. I. . (2020). Severe acute pancreatitis: capillary permeability model linking systemic inflammation to multiorgan failure. Am. J. Physiol. Gastrointest Liver Physiol. 319, G573–g583. doi: 10.1152/ajpgi.00285.2020, PMID: 32877220 PMC8087347

[B15] Li J. Gao J. Huang M. Fu X. Fu B. (2024a). Risk factors for death in patients with severe acute pancreatitis in guizhou province, China. Gastroenterol. Res. Pract. 2024, 8236616. doi: 10.1155/2024/8236616, PMID: 38590392 PMC11001474

[B16] Li X. Zhang Y. Wang W. Meng Y. Chen H. Chu G. . (2024b). An inflammation-based model for identifying severe acute pancreatitis: a single-center retrospective study. BMC Gastroenterol. 24, 63. doi: 10.1186/s12876-024-03148-4, PMID: 38317108 PMC10840143

[B17] Liţă Cofaru F. A. Eremia I. A. Nica S. Brînduşe L. A. Zărnescu N. O. Moldoveanu A. C. . (2025). Predictive value of several parameters for severity of acute pancreatitis in a cohort of 172 patients. Diagnostics (Basel) 15, 435. doi: 10.3390/diagnostics15040435, PMID: 40002586 PMC11854639

[B18] Liu Y. Qin S. Dai S. Zhou J. Wang Z. Yin G. (2025). The effect of blood glucose levels on serum triglyceride clearance in patients with hyperlipidemic acute pancreatitis. Sci. Rep. 15, 2647. doi: 10.1038/s41598-024-83342-2, PMID: 39837897 PMC11751154

[B19] Liu C. Zhou X. Ling L. Chen S. Zhou J. (2019). Prediction of mortality and organ failure based on coagulation and fibrinolysis markers in patients with acute pancreatitis: A retrospective study. Med. (Baltimore) 98, e15648. doi: 10.1097/md.0000000000015648, PMID: 31124944 PMC6571240

[B20] Lu L. Feng Y. Liu Y. H. Tan H. Y. Dai G. H. Liu S. Q. . (2022). The systemic immune-inflammation index may be a novel and strong marker for the accurate early prediction of acute kidney injury in severe acute pancreatitis patients. J. Invest. Surg. 35, 962–966. doi: 10.1080/08941939.2021.1970864, PMID: 34468253

[B21] Luo Z. Shi J. Fang Y. Pei S. Lu Y. Zhang R. . (2023). Development and evaluation of machine learning models and nomogram for the prediction of severe acute pancreatitis. J. Gastroenterol. Hepatol. 38, 468–475. doi: 10.1111/jgh.16125, PMID: 36653317

[B22] Ma Y. Yue P. Zhang J. Yuan J. Liu Z. Chen Z. . (2024). Early prediction of acute gallstone pancreatitis severity: a novel machine learning model based on CT features and open access online prediction platform. Ann. Med. 56, 2357354. doi: 10.1080/07853890.2024.2357354, PMID: 38813815 PMC11141304

[B23] Nian W. Tao W. Zhang H. (2025). Review of research progress in sepsis-associated acute kidney injury. Front. Mol. Biosci. 12. doi: 10.3389/fmolb.2025.1603392, PMID: 40718792 PMC12289498

[B24] Peng W. Li K. Song Y. Zhu Y. (2025). Early predictors for hypertriglyceridemic acute pancreatitis. Sichuan Da Xue Xue Bao Yi Xue Ban 56, 591–595. doi: 10.12182/20250360610, PMID: 40599267 PMC12207051

[B25] Potievskiy M. B. Petrov L. O. Ivanov S. A. Sokolov P. V. Trifanov V. S. Grishin N. A. . (2025). Machine learning for modeling and identifying risk factors of pancreatic fistula. World J. Gastrointest Oncol. 17, 100089. doi: 10.4251/wjgo.v17.i4.100089, PMID: 40235910 PMC11995311

[B26] Qi M. Lu C. Dai R. Zhang J. Hu H. Shan X. (2024). Prediction of acute pancreatitis severity based on early CT radiomics. BMC Med. Imaging 24, 321. doi: 10.1186/s12880-024-01509-9, PMID: 39604925 PMC11603661

[B27] Seifter J. L. Chang H. Y. (2017). Disorders of acid-base balance: new perspectives. Kidney Dis. (Basel) 2, 170–186. doi: 10.1159/000453028, PMID: 28232934 PMC5260542

[B28] Szatmary P. Grammatikopoulos T. Cai W. Huang W. Mukherjee R. Halloran C. . (2022). Acute pancreatitis: diagnosis and treatment. Drugs 82, 1251–1276. doi: 10.1007/s40265-022-01766-4, PMID: 36074322 PMC9454414

[B29] Tan Z. Li G. Zheng Y. Li Q. Cai W. Tu J. . (2024). Advances in the clinical application of machine learning in acute pancreatitis: a review. Front. Med. (Lausanne) 11. doi: 10.3389/fmed.2024.1487271, PMID: 39839637 PMC11747317

[B30] Thapa R. Iqbal Z. Garikipati A. Siefkas A. Hoffman J. Mao Q. . (2022). Early prediction of severe acute pancreatitis using machine learning. Pancreatology 22, 43–50. doi: 10.1016/j.pan.2021.10.003, PMID: 34690046

[B31] Wang J. Chen X. Ke Y. Zhu N. Zhu J. Ding H. . (2025). Clinical significance of low-density granulocytes in acute pancreatitis. Mediators Inflammation 2025, 5275081. doi: 10.1155/mi/5275081, PMID: 40689395 PMC12271696

[B32] Wang W. Sheng R. Liao S. Wu Z. Wang L. Liu C. . (2024). LightGBM is an effective predictive model for postoperative complications in gastric cancer: A study integrating radiomics with ensemble learning. J. Imaging Inform Med. 37, 3034–3048. doi: 10.1007/s10278-024-01172-0, PMID: 38940888 PMC11612084

[B33] Xiang K. Shang D. (2025). Construction of a multi-view deep learning model for the severity classification of acute pancreatitis. Discov. Med. 37, 73–92. doi: 10.24976/Discov.Med.202537192.7, PMID: 39851225

[B34] Xiao W. Liu W. Yin L. Li Y. Lu G. Liu X. . (2020). Serum hydroxybutyrate dehydrogenase as an early predictive marker of the severity of acute pancreatitis: a retrospective study. BMC Gastroenterol. 20, 393. doi: 10.1186/s12876-020-01521-7, PMID: 33218299 PMC7678267

[B35] Xu F. Hu X. Li S. L. (2023). Exploring the value of early laboratory indicators combined with pancreatitis activity scoring system in assessing the severity and prognosis of acute pancreatitis. Pak J. Med. Sci. 39, 1462–1467. doi: 10.12669/pjms.39.5.7543, PMID: 37680829 PMC10480758

[B36] Yan T. Ma Y. Wang Z. Lyu J. Wu S. Zhang C. . (2024). Calcium administration appears not to benefit acute pancreatitis patients with hypocalcemia. J. Hepatobiliary Pancreat Sci. 31, 273–283. doi: 10.1002/jhbp.1397, PMID: 38058277

[B37] Yang K. Song Y. Su Y. Li C. Ding N. (2024). Establishment and validation of an early predictive model for severe acute pancreatitis. J. Inflammation Res. 17, 3551–3561. doi: 10.2147/jir.s457199, PMID: 38855164 PMC11162219

[B38] Yuan J. Xiong J. Yang J. Dong Q. Wang Y. Cheng Y. . (2025). Machine learning-based 28-day mortality prediction model for elderly neurocritically Ill patients. Comput. Methods Programs BioMed. 260, 108589. doi: 10.1016/j.cmpb.2025.108589, PMID: 39799642

[B39] Zerem E. Kurtcehajic A. Kunosić S. Zerem Malkočević D. Zerem O. (2023). Current trends in acute pancreatitis: Diagnostic and therapeutic challenges. World J. Gastroenterol. 29, 2747–2763. doi: 10.3748/wjg.v29.i18.2747, PMID: 37274068 PMC10237108

[B40] Zhang F. (2025). Combination of red cell distribution width, plasma albumin, and serum amylase as marker of severity of infection in emergency patients with acute pancreatitis. Clin. Lab. 71, 10.7754. doi: 10.7754/Clin.Lab.2024.241031, PMID: 40497625

[B41] Zhang B. Chen L. Li T. (2025). Unveiling the effect of urinary xenoestrogens on chronic kidney disease in adults: A machine learning model. Ecotoxicol Environ. Saf. 292, 117945. doi: 10.1016/j.ecoenv.2025.117945, PMID: 39987685

[B42] Zhao X. Wang Y. Li J. Liu W. Yang Y. Qiao Y. . (2025). A machine-learning-derived online prediction model for depression risk in COPD patients: A retrospective cohort study from CHARLS. J. Affect. Disord. 377, 284–293. doi: 10.1016/j.jad.2025.02.063, PMID: 39988142

[B43] Zhou Y. Han F. Shi X. L. Zhang J. X. Li G. Y. Yuan C. C. . (2022). Prediction of the severity of acute pancreatitis using machine learning models. Postgrad Med. 134, 703–710. doi: 10.1080/00325481.2022.2099193, PMID: 35801388

[B44] Zhou L. X. Zhou Q. Gao T. M. Xiang X. X. Zhou Y. Jin S. J. . (2024). Machine learning predicts acute respiratory failure in pancreatitis patients: A retrospective study. Int. J. Med. Inform 192, 105629. doi: 10.1016/j.ijmedinf.2024.105629, PMID: 39321493

[B45] Zou Y. Li K. Geng P. (2025). Analysis of influencing factors of acute pancreatitis complicated with persistent inflammation and construction of a prediction model. Pancreas 54, e873–e879. doi: 10.1097/mpa.0000000000002526, PMID: 40554769

