# Supplementary material for: Predicting the acute pancreatitis severity with multi-machine learning models: constructing an online prediction platform
Source: Front Cell Infect Microbiol. 2026 Feb 27;16:1760036. doi: 10.3389/fcimb.2026.1760036 (PMC12982339; doi:10.3389/fcimb.2026.1760036)
Supplement: Supplementary file 1 [file DataSheet1.docx]

Supplementary Material

**Table S1.** Baseline clinical characteristics of patients.

| Variable | Training cohort  (n = 902) | Test cohort  (n = 387) |
| --- | --- | --- |
| Sex |  |  |
| Male | 575 (63.7%) | 262 (67.7%) |
| Female | 327(36.3%) | 125(32.3%) |
| White Blood Cell Count (WBC) (10^9^/L) | 12.34(9.18, 15.98) | 11.94(9.12, 15.15) |
| Red Blood Cell Count (RBC) (10^12^/L) | 4.68(4.23, 5.14) | 4.73±0.79 |
| Hemoglobin (Hb) (g/L) | 141 (126,157) | 141.75 ± 24.03 |
| Platelet Count (PLT) (10^9^/L) | 229 (185, 283) | 232 (190, 287) |
| Neutrophils Percentage (NEU%) (%) | 81.90 (73.48, 87.9) | 81.20(73.7, 87) |
| Neutrophils Absolute Count (NEU#) (10^9^/L) | 9.91 (7.11, 13.42) | 9.59 (6.81, 12.82) |
| Lymphocytes Percentage (LYM%) (%) | 10.8 (6.60, 17.25) | 11.1 (6.9, 17.9) |
| Lymphocytes Absolute Count (LYM#) (10^9^/L) | 1.29(0.86, 1.84) | 1.32(0.88, 1.89) |
| Monocytes Percentage (MON%) (%) | 5.80 (4.4, 7.5) | 5.8 (4.7, 7.5) |
| Monocytes Absolute Count (MON#) (10^9^/L) | 0.7 (0.5, 0.94) | 0.69 (0.52, 0.93) |
| Eosinophils Percentage (EOS%) (%) | 0.3 (0.1, 1.1) | 0.4 (0.1, 1.1) |
| Eosinophils Absolute Count (EOS#) (10^9^/L) | 0.04 (0.01, 0.12) | 0.05 (0.01, 0.12) |
| Basophil Percentage (BAS%) % | 0.20 (0.1, 0.4) | 0.2 (0.1, 0.4) |
| Basophil Absolute Count (BAS#) (10^9^/L) | 0.03 (0.02, 0.04) | 0.03(0.02, 0.04) |
| Hematocrit (HCT) (%) | 41.7 (37.9, 45) | 41.65 ± 6.21 |
| Mean Corpuscular Volume (MCV) (fL) | 89.8(85.7, 93.2) | 89.6(85.7, 93.8) |
| Mean Corpuscular Hemoglobin (MCH) (pg) | 30.5 (29.1, 32) | 30.5(29.2, 31.8) |
| Mean Corpuscular Hemoglobin Concentration (MCHC) (g/L) | 336 (326, 348) | 336 (326, 350) |
| Red Cell Distribution Width - Coefficient of Variation (RDW-CV) (%) | 13 (12, 14) | 13 (12,14) |
| Red Cell Distribution Width - Standard Deviation (RDW-SD) (fL) | 42.2 (39.6, 45) | 41.8 (39.6, 45) |
| Plateletcrit (PCT) (%) | 0.23 (0.19, 0.28) | 0.24 (0.2, 0.29) |
| Mean Platelet Volume (MPV) (fL) | 10.1 (9.5, 10.9) | 10.1 (9.5, 10.9) |
| Platelet Distribution Width (PDW) (%) | 11.5 (10.2, 13.1) | 11.7 (10.2, 13.2) |
| Platelet Large Cell Ratio (P-LCR) (%) | 25.8 (20.9, 31.9) | 26.5 (21.2, 32.1) |
| CRP (mg/L) | 25.48 (5.78, 89.48) | 25.64 (4.49, 78.84) |
| Total Protein (TP) (g/L) | 73.4 (67.98, 77.53) | 72.6 (68.4, 77) |
| Albumin (ALB) (g/L) | 41.45 (36.8, 44.73) | 41.3(37.7, 44.2) |
| Globulin (GLOB) (g/L) | 31.85 (28.50, 35.9) | 31.8 (28.3, 36) |
| Albumin/Globulin Ratio (A/G) | 1.28 (1.06, 1.49) | 1.28 (1.08, 1.46) |
| Total Bilirubin (TBIL) (μmol/L) | 13.35 (8.2, 23.43) | 12.7 (8.4, 21.8) |
| Indirect Bilirubin (IBIL) (μmol/L) | 6.6 (4.4, 10.33) | 6.9 (4.5, 10.1) |
| Direct Bilirubin (DBIL) (μmol/L) | 5.5 (3.1, 11.70) | 5.5 (3.1, 10) |
| Alanine Aminotransferase (ALT) (U/L) | 29.55 (16.08, 64.18) | 30 (17.6, 76.2) |
| Aspartate Aminotransferase (AST) (U/L) | 29.6 (20.1, 68.15) | 32.5 (19.8, 85.1) |
| AST/ALT Ratio | 1.15 (0.8, 1.77) | 1.13 (0.77, 1.68) |
| Gamma-Glutamyl Transferase (GGT) (U/L) | 88.35 (45.83, 184) | 95 (51.4, 201) |
| Alkaline Phosphatase (ALP) (U/L) | 81 (66, 105) | 80.5 (65.4, 109) |
| Creatinine (Cr) (μmol/L) | 73 (61, 87) | 73 (61, 87) |
| Urea (mmol/L) | 4.8(3.6,6.5) | 4.7(3.7,6.2) |
| Carbon Dioxide Combining Power (CO₂-CP) (mmol/L) | 23.1 (20.8, 25.33) | 23.06 (20.2, 25) |
| Creatine Kinase (CK) (U/L) | 95 (62.88, 156) | 92 (65, 153) |
| Creatine Kinase-MB (CK-MB) (ng/ml) | 14.93 (10.99, 21.43) | 14.9 (10.6, 21.7) |
| Lactate Dehydrogenase (LDH) (U/L) | 239 (191, 327.5) | 234 (188, 321) |
| Alpha-Hydroxybutyrate Dehydrogenase (α-HBDH) (U/L) | 169.69 (139, 224.61) | 167 (135, 221) |
| AMY (U/L) | 334.5 (124.63, 1019) | 335 (100, 916) |
| Potassium (K) (mmol/L) | 3.85 (3.6, 4.12) | 3.81 (3.61, 4.1) |
| Chloride (Cl) (mmol/L) | 101.4 (98, 104.1) | 101.4 (98.4, 103.9) |
| Sodium (Na) (mmol/L) | 138.6 (135.8, 141) | 139 (136, 141) |
| Calcium (Ca) (mmol/L) | 2.23 (2.11, 2.31) | 2.24 (2.12, 2.32) |
| Triglycerides (TG) (mmol/L) | 2.99 (1.17, 8.38) | 3.53 (1.13, 9.74) |
| Cholesterol (CHOL) (mmol/L) | 4.93 (3.93, 6.26) | 5.07 (4.14, 6.51) |
| Low-Density Lipoprotein Cholesterol (LDL-C) (mmol/L) | 2.22 (1.34, 2.87) | 2.35 (1.45, 2.93) |
| Prothrombin Time (PT) (S) | 13 (12.3, 13.9) | 13 (12.3, 13.8) |
| International Normalized Ratio (INR) | 1.03 (0.97, 1.12) | 1.03 (0.97, 1.11) |
| Activated Partial Thromboplastin Time (APTT) (S) | 33.4 (30.1, 37.6) | 33.8 (30.3, 37.7) |
| Fibrinogen (FIB) (g/L) | 3.77 (3.05, 5.13) | 3.81 (3.01, 4.96) |
| Thrombin Time (TT) (S) | 16.5 (15.5, 17.8) | 16.5 (15.6, 18.2) |
| Glucose (Glu) (mmol/L) | 7.77 (6.3, 10.07) | 7.93 (6.43, 10.25) |





Figure S1. Relationship between features and SAP and (A)PT, (B)ALB, (C)CRP, (D)MCH, (E)APTT, (F)Hb and (G)CO_2_-CP.
